# Supplementary material for: Inherently Fluorescent Peanut-Shaped Polymersomes for Active Cargo Transportation
Source: Pharmaceutics. 2023 Jul 20;15(7):1986. doi: 10.3390/pharmaceutics15071986 (PMC10385398; doi:10.3390/pharmaceutics15071986)
Supplement: Supplementary file 1 [file pharmaceutics-15-01986-s001.zip › pharmaceutics-2492475-supplementary.pdf]

Supplementary Materials

Inherently Fluorescent Peanut-shaped Polymersomes for Active Cargo Transportation

Jianhong Wang [1,†], Yingtong Luo [1,†], Hanglong Wu [1], Shoupeng Cao [2], Loai K. E. A. Abdelmohsen [1], Jingxin Shao [1,*] and Jan C. M. van Hest [1,*]

Experimental

1. Instruments

1.1. Nuclear Magnetic Resonance Spectroscopy (NMR):

Routine proton nuclear magnetic resonance (^{1}H NMR) measurements were recorded on a Bruker AV 400 MHz Ultrashield™ spectrometer, $CDCl_3$ was selected as the solvent and tetramethylsilane (TMS) as the internal standard for ^{1}H NMR.

1.2. Gel Permeation Chromatography (GPC):

The molecular weights (M_n/M_w) and dispersity index (PDI) of the block polymers were measured by using a Prominence-I GPC system (Shimadzu) with a PL gel 5 μm mixed D (Polymer Laboratories) and equipped with a RID-20A differential refractive index detector. Polystyrene standards were used for calibration. THF was used as an eluent, with a flow rate of 1 mL/min.

1.3. Dynamic Light scattering (DLS):

The hydrodynamic size and dispersity index (PDI) of the nanoparticles were determined by a Malvern instruments Zetasizer (model Nano ZSP) equipped with a 633 nm He-Ne laser and avalanche photodiode detector. Zetasizer software was further used to analyze the data.

1.4. Transmission Electron Microscopy (TEM):

TEM images were recorded by a FEI Tecnai 20 (type Sphera) at 200 kV. Samples for TEM were prepared by dropping 20 μL of sample solution onto a Cressington 208 carbon-coated copper grid for 40 seconds. The samples were dried at room temperature.

1.5. Cryogenic transmission electron microscopy (cryo-TEM):

Experiments were measured using the cryo-Titan (Thermo Fisher Scientific) equipped with a field emission gun and operated at 300 kV acceleration voltage, an autoloader station and a post-column Gatan energy filter. Samples for cryo-TEM were prepared by glow-discharging the grids (cryo-TEM: Lacey carbon coated, R2/2, Cu, EM sciences) in a Cressington 208 carbon coater for 40 seconds. Then, 3 μL of the sample's solution was pipetted on the grid and blotted in a Vitrobot MARK III at 100% humidity. The grid was blotted for 3.5 seconds (offset -3) and directly plunged and frozen in liquid ethane. Cryo-TEM images were collected with zero loss energy filtering mode (Gatan GIF 2002, 20eV energy slit) on a CCD camera (Gatan model 794).

1.6. Two Photon-Confocal Laser Scanning Microscopy (TP-CLSM):

Fluorescent images were observed and recorded using a CLSM (Leica TCS SP5X) equipped with two-photon laser source (Chameleon Vision, Coherent, USA).

1.7. Microplate Reader:

Cell viability was evaluated via a microplate reader (Safire2, TECAN). The reaction progress was monitored via the fluorescent signal from fluorescein on the Spark ® 10M microplate reader (TECAN).

1.8. UV-vis spectroscopy:

UV-vis absorbance was determined by UV-vis spectroscopy (V-650, JASCO) using a 1 mL black quartz cuvette.

1.9. NanoSight Tracking Analysis (NTA):

Nanosight Tracking Analysis was performed on a Nanosight NS300 equipped with laser channel (488 nm) and sCMOS camera. To analyze the autonomous motion under laser (405 nm) irradiation, the NanoSight was equipped with an Electron Multiplication Charge Coupled Device and an external light source (405 nm, BeamQ Lasers) was used.

2. Movement analysis:

NTA was used to study the autonomous motion of peanut polymersomes in absence and presence of laser irradiation and their mean squared displacement (MSD) was calculated, following previously published procedures[31,37–39]. The motion of peanut polymersomes upon 405 nm laser irradiation was measured as a function of output laser power by tracking both the X and Y coordinates of at least 25 particles for 30 seconds. MSD curves were extracted from NTA recorded trajectories using the following equation:

$$\boldsymbol{MSD} = [\Delta \mathbf{r}^2(\mathbf{t})] = \left[\frac{\mathbf{1}}{\boldsymbol{N}}\sum_{\boldsymbol{i=0}}^{\boldsymbol{N}}\big(\boldsymbol{r_i}(\boldsymbol{t}) - \boldsymbol{r_i}(\mathbf{0})\big)^{\mathbf{2}}\right]$$

where r = radius and t = sampling time and MSD(t) = 2dD, where D = diffusion coefficient and d = dimensionality (NTA measurements have dimension d = 2). The equation MSD = (4D)Δt +(v^2)(Δt^2) was used to fit the MSD curves. From the fitting of the MSD curves, the average particle velocity was extracted. According to the particle diffusion coefficient, as described by Galestonian's diffusiophoretic model, a particle undergoing Brownian motion will display a linear MSD over time with the slope determined by the diffusion coefficient $D = KBT/(6\pi\eta R)$. From this model, if the particles are in Brownian motion, the linear component of the MSD, according to the equation MSD = (4D)Δt, can be extracted.

3. Synthesis routes

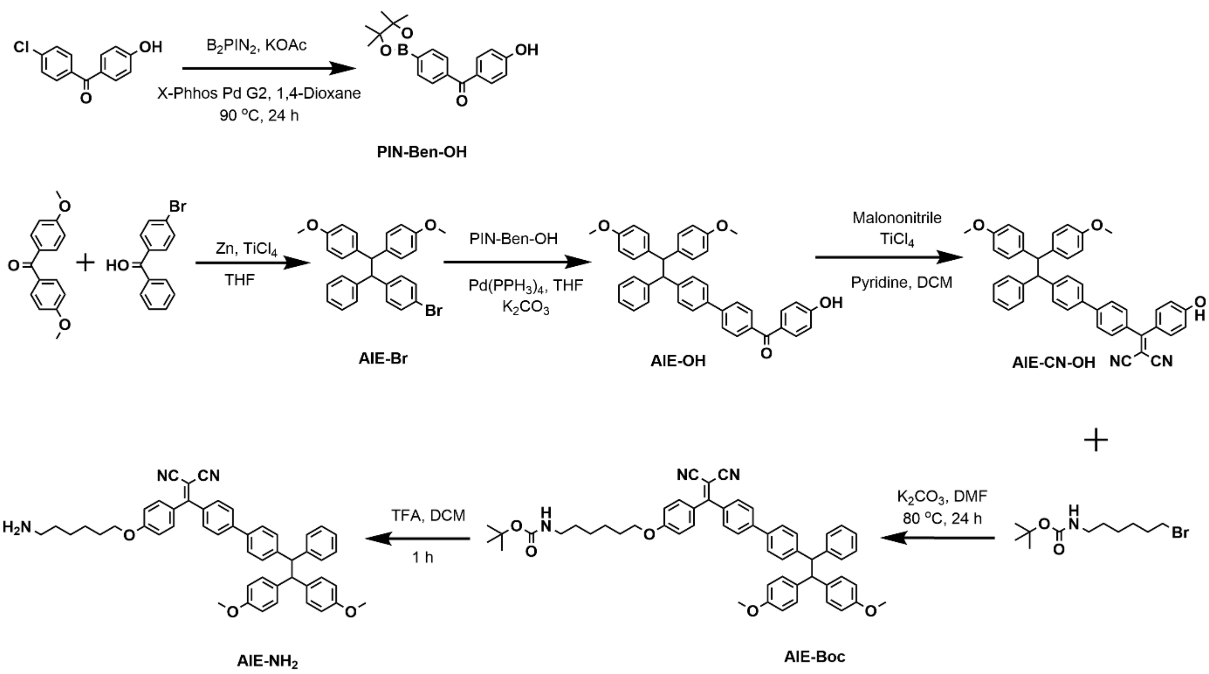

Figure S1. Synthetic route toward AIE-NH_2 (**1**).

This NH_2-modified AIE molecule **1** (AIE-gen **1**) was synthesized according to our previously reported literature procedure, which are briefly described as follows[29].

Compound PIN-Ben-OH [40]**:** Under nitrogen environment at room temperature, X-Phos Pd G2 precatalyst (190 mg, 0.24 mmol, 0.05 equiv), bis(pinacolato)diboron (B_2Pin_2) (7.32 g, 28.8 mmol, 6.00 equiv), potassium acetate (KOAc, 2.83 g, 28.8 mmol, 6.00 equiv), and 4-chloro-4-hydroxybenzophenone (1.11 g, 4.8 mmol, 1.00 equiv) were added into a 500 mL round-bottom flask vial equipped with a stir bar. Thereafter, anhydrous 1,4-dioxane (250 mL) was added, and the vial was sealed with a screw cap and placed in a mantle heated to 90 °C. After being stirred (500 rpm) for 24 h, the formed golden-brown mixture was allowed to cool to room temperature, diluted with EtOAc (100 mL), suction-filtered through a short plug of silica gel, after which the plug was rinsed with EtOAc. This resulted in a pale-yellow filtrate which was concentrated and separated via silica gel chromatography. The crude product was used for the synthesis of AIE-OH without further purification.

Compound AIE-Br [41]**:** Briefly, bis(4-methoxyphenyl) methanone (9.73 g, 40.0 mmol), 4-bromo-benzophenone (12.54 g, 48.0 mmol), and Zn powder (13 g, 200 mmol) were dissolved in dry THF pre-cooled on an ice-bath (500 mL) under argon atmosphere. While stirring, titanium tetrachloride ($TiCl_4$, 17.6 mL, 160 mmol) was slowly added into the reaction mixture. Subsequently, the mixture was heated to 80 °C. After 24 h, the reaction mixture was left to cool down to room temperature. Afterwards, the reaction was quenched by the addition of 250 mL 10% aqueous K_2CO_3, and the mixture was filtered to remove insoluble materials and washed with CH_2Cl_2. The organic layer was dried with anhydrous $MgSO_4$ and filtered. The CH_2Cl_2 solution was removed by evaporation. The crude product was purified by column chromatography to give 9.62 g (20.4 mmol) of AIE-Br as a light yellow solid in 51% yield.^{1}H NMR (400 MHz, $CDCl_3$) δ (ppm): 7.21 (d, J = 8.3 Hz, 2H), 7.16 – 7.04 (m, 3H), 7.05 – 6.96 (m, 2H), 7.01 – 6.85 (m, 6H), 6.70 – 6.59 (m, 4H), 3.75 (d, J = 10.9 Hz, 6H).

Compound AIE-OH [42]**:** Compound PIN-Ben-OH (972 mg, 3.0 mmol), AIE-Br (1550 mg, 3.3 mmol), potassium carbonate (4140 mg, 30 mmol), THF (90 mL)/water (30 mL), and Pd(PPh_3)$_4$ (3 %), were degassed and charged with nitrogen. The reaction mixture was then stirred at 60 °C for 12 h. After cooling down the reaction mixture to ambient temperature, it was extracted with dichloromethane and washed with water. The dichloromethane

layer was separated and dried over $MgSO_4$. After dichloromethane evaporation by rotary evaporation, the crude product was purified by column chromatography on silica gel using n-hexane/dichloromethane (1/1, v/v) as the eluent to afford AIE-OH as a yellow solid (1234 mg, 71 % yield). ^{1}H NMR (400 MHz, $CDCl_3$) δ (ppm): 7.86 – 7.76 (m, 4H), 7.69 – 7.62 (m, 2H), 7.43 – 7.37 (m, 2H), 7.19 – 7.06 (m, 7H), 7.01 – 6.89 (m, 6H), 6.72 – 6.61 (m, 4H), 3.74 (s, 6H). ^{13}C NMR (100 MHz, $CDCl_3$) δ 195.40, 159.89, 158.22, 158.13, 144.40, 144.14, 140.68, 138.57, 137.21, 136.55, 136.26, 132.81, 132.63, 132.60, 132.00, 131.44, 130.43, 130.36, 127.79, 126.54, 126.41, 126.23, 115.20, 113.16, 113.03, 77.34, 77.02, 76.70, 55.13, 55.11. MALDI TOF-MS: m/z calculated for $[M]^+$ $C_{41}H_{32}O_4$, 588.23, found 588.24.

Compound AIE-CN-OH [42]**:** To a solution of compound AIE-OH (588 mg, 1.0 mmol) and malononitrile (198 mg, 3.0 mmol) in dichloromethane (100 mL) was slowly added titanium tetrachloride (0.4 mL, 3.5 mmol) while cooling with an ice-bath. After the reaction mixture was stirred for 30 min, pyridine (0.3 mL, 3.5 mmol) was injected and stirred for another 30 min. Then the mixture was heated to 40 °C for 4 h. After the mixture was cooled down to room temperature, the reaction was quenched using water (30 mL) and the mixture was extracted with dichloromethane. The collected organic layer was washed with brine, dried over Na_2SO_4 and concentrated under reduced pressure. The residue was purified by column chromatography using n-hexane/dichloromethane (1/1 ~ 1/2, v/v) as eluent to give the desired product AIE-CN-OH as a red solid (534 mg, yield 84 %). ^{1}H NMR (400 MHz, $CDCl_3$) δ (ppm): 7.72 – 7.63 (m, 2H), 7.51 – 7.45 (m, 2H), 7.45 – 7.37 (m, 4H), 7.17 – 7.02 (m, 7H), 7.01 – 6.86 (m, 6H), 6.71 – 6.62 (m, 4H), 3.75 (s, 6H). ^{13}C NMR (100 MHz, $CDCl_3$) δ (ppm): 174.19, 160.27, 158.26, 158.18, 145.06, 144.90, 144.04, 140.89, 138.44, 136.53, 136.18, 136.16, 134.74, 133.27, 132.71, 132.63, 132.60, 132.10, 131.42, 131.26, 128.18, 127.81, 126.97, 126.37, 126.28, 115.89, 113.16, 113.04, 78.24, 77.34, 77.02, 76.70, 55.14, 55.11. MALDI TOF-MS: m/z calculated for $[M]^+$ $C_{44}H_{32}N_2O_3$, 636.24, found 636.26.

Compound AIE-Boc [43]**:** A mixture of AIE-CN-OH (1.75 g, 2.76 mmol), 6-bromo-1-hexanol (500 mg, 2.76 mmol), and K_2CO_3 (950 mg, 6.90 mmol) in DMF (100 mL) was stirred at 80 °C overnight under nitrogen atmosphere. The reaction mixture was cooled to room temperature, filtered through a pad of celite, and washed with CH_2Cl_2. The solvent was then removed via rotary evaporation and re-dissolved in CH_2Cl_2. The DCM solution was then washed with water, dried with $MgSO_4$ and filtered. The solvent was removed by evaporation. The crude mixture was purified using column chromatography on silica gel using n-hexane/dichloromethane (4/1 ~ 2/1, v/v). The product was obtained as a red solid (2.00 g, 89% yield). ^{1}H NMR (400 MHz, $CDCl_3$) δ (ppm): 7.71 – 7.63 (m, 2H), 7.49 – 7.43 (m, 4H), 7.43 – 7.37 (m, 2H), 7.12 (m, 5H), 7.05 (m, 2H), 7.00 – 6.90 (m, 6H), 6.70 – 6.61 (m, 4H), 4.51 (s, 1H), 4.03 (t, J = 6.4 Hz, 2H), 3.75 (d, J = 1.3 Hz, 6H), 3.13 (q, J = 6.6 Hz, 2H), 1.82 (m, 2H), 1.50 (m, 4H), 1.44 (s, 9H), 1.26 (m, 2H). ^{13}C NMR (100 MHz, $CDCl_3$) δ ppm: 173.94, 163.08, 158.26, 158.17, 155.99, 144.92, 144.85, 144.04, 140.86, 138.43, 136.57, 136.16, 134.86, 133.03, 132.62, 132.59, 132.08, 131.42, 131.24, 127.80, 126.92, 126.36, 114.68, 113.14, 113.02, 78.23, 77.33, 77.01, 76.70, 68.27, 55.12, 55.10, 40.47, 30.05, 28.97, 28.43, 26.49, 25.70. MALDI TOF-MS: m/z calculated for $[M]^+$ $C_{55}H_{53}N_3O_5$, 835.40, found 835.42.

Compound AIE-NH$_2$: AIE-Boc (1.67 g, 2 mmol) was dissolved in DCM (10 mL) and immersed in an ice bath. Then, 5 mL trifluoroacetic acid was added dropwise under nitrogen atmosphere. The reaction mixture was allowed to warm to room temperature and reacted for another 1 h. The solvent was removed, and the crude mixture was dissolved in CH_2Cl_2, washed with water, and dried with $MgSO_4$. Then the CH_2Cl_2 solvent was removed via evaporation and the product was obtained as a red solid (1.38 g, 94% yield). ^{1}H NMR (400 MHz, $CDCl_3$) δ (ppm): 7.65 (d, J = 8.1 Hz, 2H), 7.49 – 7.36 (m, 6H), 7.15 – 7.02 (m, 7H), 7.00 – 6.87 (m, 6H), 6.65 (m, 4H), 4.03 (t, J = 6.2 Hz, 2H), 3.74 (d, J = 1.9 Hz, 6H), 3.10 – 3.00 (m, 2H), 1.81 (m, 2H), 1.50 – 1.38 (m, 4H), 1.27 (m, 2H). ^{13}C NMR (100 MHz, $CDCl_3$) δ (ppm): 174.54, 163.08, 158.23, 158.15, 145.15, 144.93, 144.02, 140.90, 138.47, 136.51, 136.23, 136.19, 134.68, 133.05, 132.64, 132.60, 132.11, 131.42, 131.30, 128.01, 127.82, 126.96, 126.38, 126.29, 114.72, 113.19, 113.06, 77.85, 77.34, 77.02, 76.70, 67.91, 55.14, 40.53, 28.56, 27.60, 27.25, 25.28. MALDI TOF-MS: m/z calculated for $[M]^+$ $C_{50}H_{45}N_3O_3$, 735.35, found 735.34.

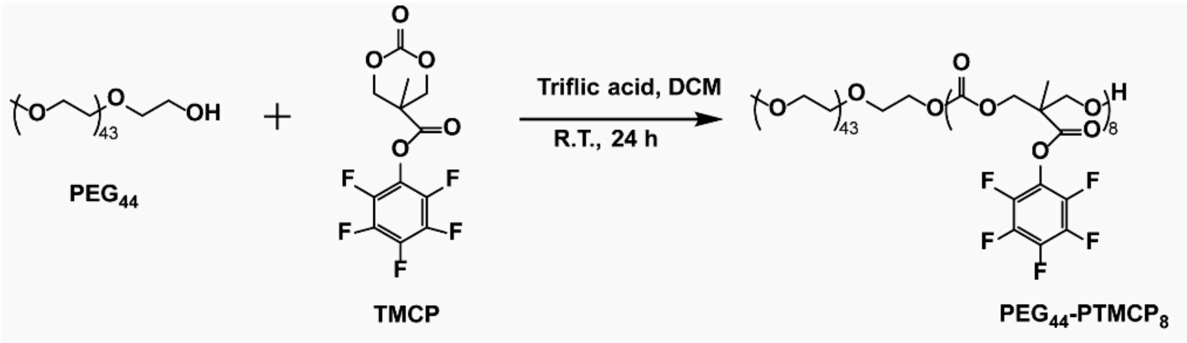

Figure S2. Synthetic route of PEG_{44}-$PTMCP_8$.

The AIE moiety pendant block copolymers were synthesized according to our previously reported literature procedures [29].

Synthesis of TMCP: A 250 mL round bottom flask was charged with 2,2-bis(hydroxymethyl)propionic acid (bis-MPA) (3.00 g, 22 mmol), bis-(pentafluorophenyl)carbonate (21.70 g, 55 mmol, 2.5 eq.), CsF (0.7 g, 4.6 mmol, 0.2 eq.), and 120 mL of anhydrous tetrahydrofuran (THF). After 21 h, the solvent THF was removed in vacuum. The residue was re-dissolved in dichloromethane, and after 10 min, a byproduct precipitated which was removed by filtration. The filtrate was extracted with sodium bicarbonate and water, and then dried with $MgSO_4$. The solvent was evaporated in vacuum and the product was recrystallized from an ethyl acetate/hexane mixture to give TMC-OPh_5(TMCP) as a white crystalline powder. Yield: 5.13 g (70 % yield). ^{1}H NMR (400 MHz, $CDCl_3$), δ ppm: 4.85 (d, J = 10.8 Hz, 2H,), 4.36 (d, J = 10.8 Hz, 2H,), 1.55 (s, 3H). ^{19}F NMR (376 MHz, $CDCl_3$), δ ppm: 154.0~154.1 (m, 2F), 157.3 (t, 1F), 162.4~162.5 (m, 2F).

Ring-opening polymerization (ROP) of TMCP to obtain PEG_{44}-$PTMCP_8$: The synthesis of PEG_{44}-$PTMCP_8$ was performed according to a reported procedure [44]. Monomethyl-PEG-OH macro-initiator 2 kDa (984 mg, 0.5 mmol, 1.0 equiv) and dry TMCP (1304 mg, 4 mmol, 8 equiv) were added into a round bottom flask. Under argon flow, 4 mL dichloromethane (DCM) (1 M with respect to TMCP) was added into the flask. The TMCP only partially dissolves at this concentration. Triflic acid (75 μL, 1 mmol, 2 equiv) was added to the stirring solution. As the reaction proceeded, the undissolved TMCP slowly went into solution. The reaction was monitored by ^{1}H NMR. Once the reaction was complete, the polymer was precipitated into hexanes. The crude polymer was then re-dissolved in minimal amount of DCM and precipitated into diethyl ether, isolated, and freeze dried to obtain the product, and kept in the freezer until being used. Copolymer composition was calculated by using the protons of PEG (3.65-3.7 ppm), the terminal methyl unit (singlet at 3.38 ppm), TMCP CH_2 (singlet, 4.44-4.48), and TMCP CH_3 (singlet, 1.48-1.50). GPC (RI): M_n (PDI) = 4.38 kDa (1.10).

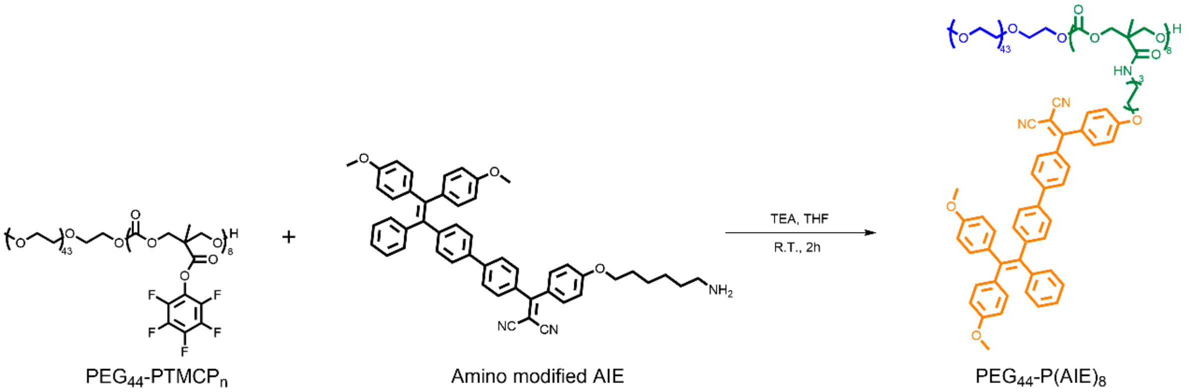

Figure S3. Synthetic route of PEG_{44}-$P(AIE)_8$.

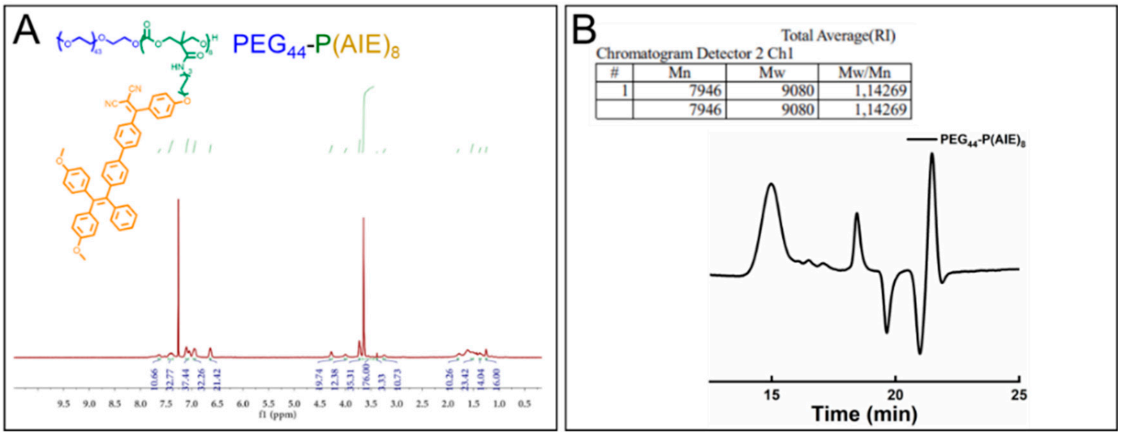

Figure S4. Characterization of amphiphilic block copolymer PEG_{44}-$P(AIE)_8$ by ^1H NMR spectroscopy (A) and GPC (B).

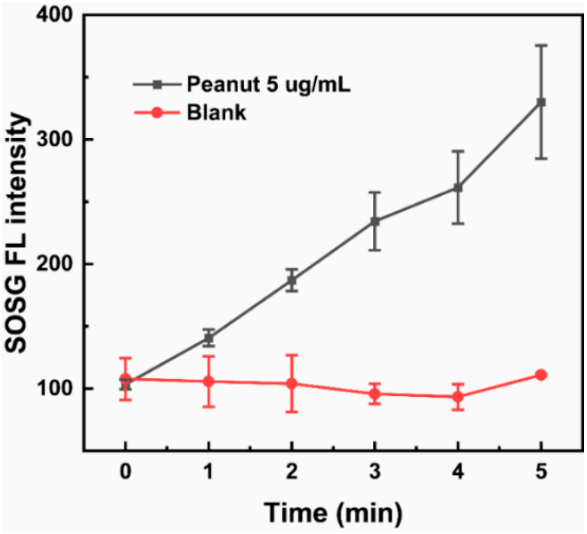

Figure S5. *Ex vitro* generation of $^1O_2$ by peanut polymersomes with (dark grey) / without (red) 405 nm laser irradiation evaluated by the indicator, singlet oxygen sensor green (SOSG, 10 μM).

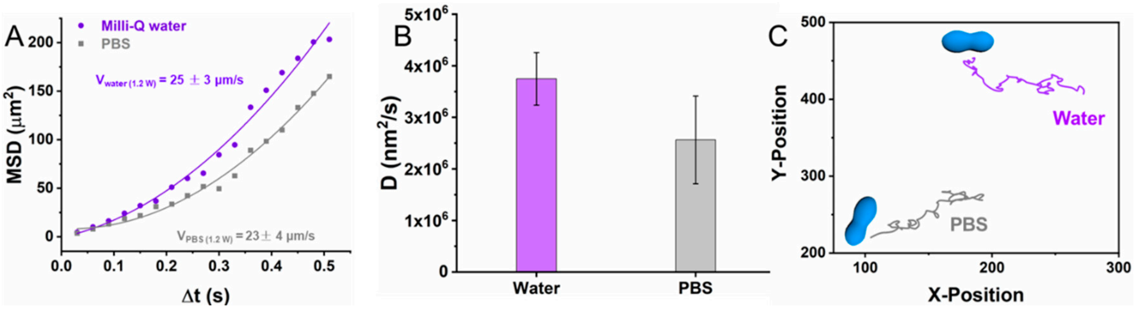

Figure S6. Comparison in motion performance of peanut polymersomes in Milli-Q water and PBS buffer under 405 nm laser irradiation. (A) Average MSD of peanut polymersomes in Milli-Q water (purple) and PBS buffer (grey). (B) Diffusion coefficient of peanut polymersomes in Milli-Q water and PBS buffer upon 405 nm laser irradiation, respectively. (C) Representative motion trajectory of peanut polymersomes in Milli-Q water and PBS buffer.

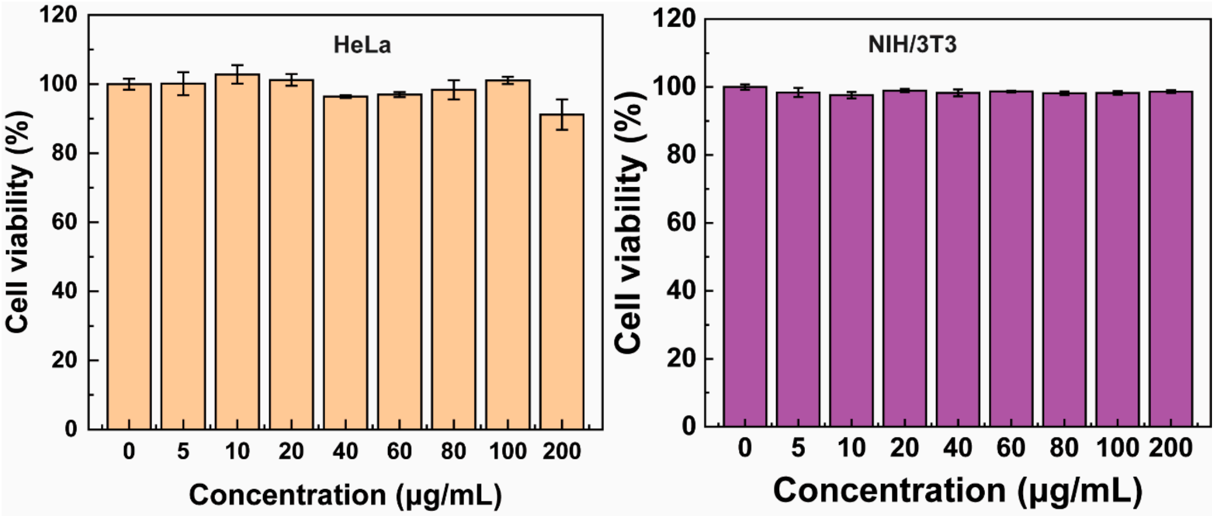

Figure S7. Cytotoxicity test of peanut polymersomes after incubation with HeLa cells (Left) and NIH/3T3 cells (Right) for 24 h.

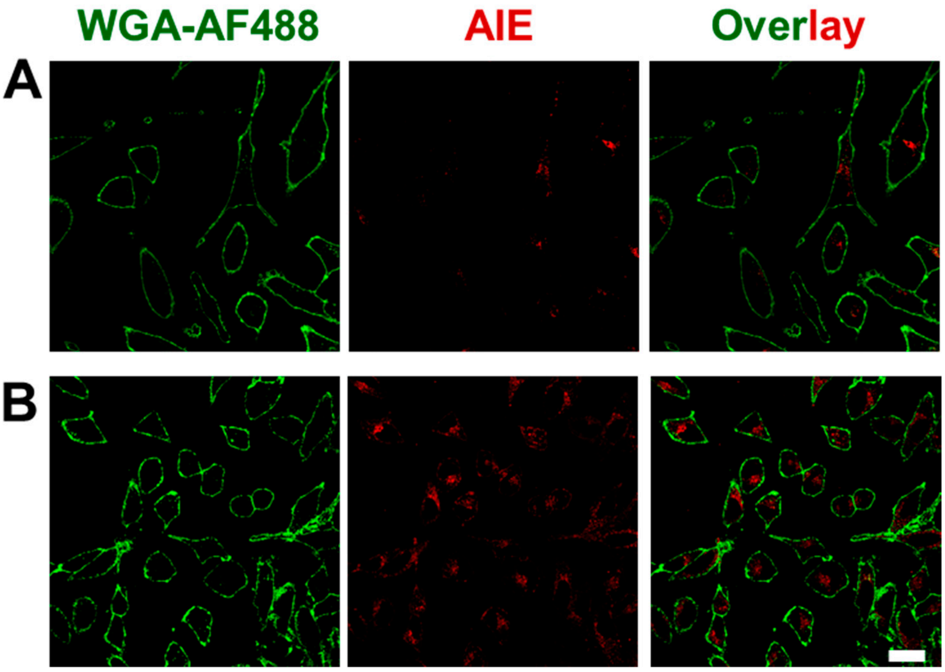

Figure S8. Characterization of enhanced cellular uptake behavior of peanut polymersomes (20 μL) towards HeLa cells. CLSM images of HeLa cells without (A) or with (B) light irradiation. Scale bar = 30 μm.

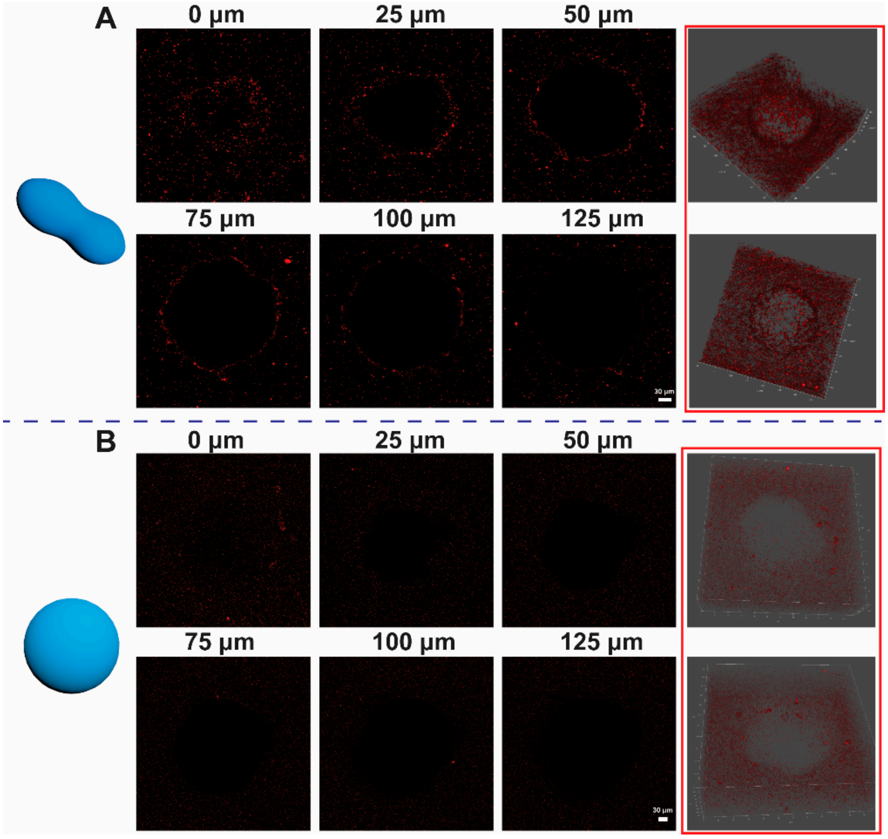

Figure S9. Enhanced penetration towards 3D tumor spheroids. (A) Z-scanning CLSM images of 3D tumor spheroids towards peanut polymersomes after irradiating with light (405 nm, 5 min). (B) Z-scanning confocal images of 3D tumor spheroids to investigate the cell uptake behavior of spherical polymersomes. In the red box on the right are 3D reconstruction images (From LAS X Life Science Microscope Software). All scale bars = 30 μm.

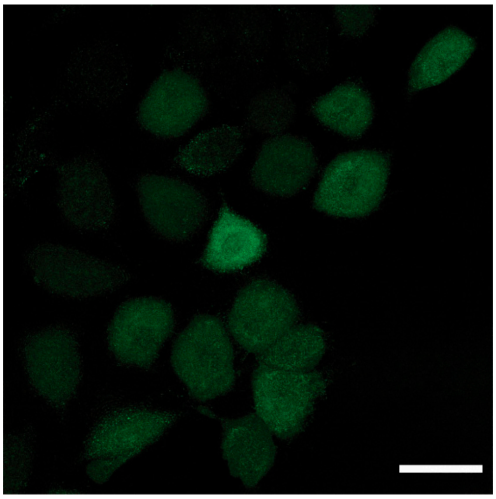

Figure S10. ROS generation in HeLa cells tested by the 2,7-dichlorodihydrofluorescein diacetate (DCF-DA) assay. Scale bar = 30 μm.

4. Supplementary videos

Video S1. Nanoparticle tracking video of spherical polymersomes under light irradiation (405 nm, 1.2 W)

Video S2. Nanoparticle tracking video of peanut polymersomes without light irradiation (405 nm, 0 W)

Video S3. Nanoparticle tracking video of peanut polymersomes under light irradiation (405 nm, 1.2 W)
